# IN-PERSON AND TECHNOLOGY-BASED SOCIAL INTERACTION IN OLDER ADULTS DURING THE COVID-19 PANDEMIC 2022

**DOI:** 10.1093/geroni/igad104.2669

**Published:** 2023-12-21

**Authors:** Jennifer Peszka, Anne Goldberg, Peter Gess, Noura Mussallam, Luke Simpson, Laura Spradley

**Affiliations:** Hendrix College, Conway, Arkansas, United States; Hendrix College, Conway, Arkansas, United States; Hendrix College, Conway, Arkansas, United States; Hendrix College, Conway, Arkansas, United States; Hendrix College, Conway, Arkansas, United States; UAMS, Little Rock, Arkansas, United States

## Abstract

Social support is an important factor in the health and well-being of older adults. Early COVID-19 lockdowns disrupted in-person social interactions but presumably increased technology-based social connection. This work examines social interactions during Spring of 2022 in older Arkansans with widely available vaccines and waning lockdowns to investigate current in-person and online social interactions. During Spring 2022, 603 older Arkansans (65+) (86.5% White, non-Hispanic, 65.9% women) completed the 20 question self-report automated survey (text or phone). Results indicated 27.6% continued to curtail in-person interactions significantly; however, 63% engaged in in-person social interactions outside their home more than once a week. Video chatting pre-pandemic was prevalent (40% video chatting multiple times a week); still, 20.6% never did. 22.8% reported increased video chatting during the pandemic, but surprisingly, 37.3% reported a decline. 70% felt socially connected during online interactions. Only 78% reported being satisfied with their social connection compared to 93% pre-pandemic. These findings suggest that while many have re-established in-person social interactions, around one-quarter of participants continue to curtail in-person interaction and report social connection dissatisfaction. Social technology alternatives were increased by about one quarter of participants and were reported to lead to feelings of social connection when used, and yet a decline in this usage was reported across 40% of these participants during the pandemic. These data support access to technology-based social interactions through expansion and improvement of older adult friendly virtual platforms and internet access which could help improve quality and quantity of social connection and reduce social isolation.

